# Cariogenic Biofilms: Development, Properties, and Biomimetic Preventive Agents

**DOI:** 10.3390/dj9080088

**Published:** 2021-08-03

**Authors:** Frederic Meyer, Joachim Enax, Matthias Epple, Bennett T. Amaechi, Barbara Simader

**Affiliations:** 1 Research Department, Dr. Kurt Wolff GmbH & Co. KG, Johanneswerkstr. 34-36, 33611 Bielefeld, Germany; joachim.enax@drwolffgroup.com (J.E.); barbara.simader@drwolffgroup.com (B.S.); 2 Inorganic Chemistry and Center for Nanointegration Duisburg-Essen (CeNIDE), University of Duisburg-Essen, Universitaetsstr. 5-7, 45117 Essen, Germany; matthias.epple@uni-due.de; 3 Department of Comprehensive Dentistry, School of Dentistry, University of Texas Health San Antonio, 7703 Floyd Curl Drive, San Antonio, TX 78229-3900, USA; amaechi@uthscsa.edu

**Keywords:** amino acids, calcium carbonate, calcium phosphates, hydroxyapatite, pH value

## Abstract

Oral biofilms will build up within minutes after cleaning of the dental hard tissues. While the application of remineralizing agents is a well-known approach to prevent dental caries, modern oral care products offer also additional active agents to maintain oral health. Human saliva contains many different organic and inorganic compounds that help to buffer organic acids produced by cariogenic microorganisms. However, most oral care products only contain remineralizing agents. To improve the benefit of those products, further active ingredients are needed. Books, review articles, and original research papers were included in this narrative review. Putting all these data together, we give an overview of oral biofilms and active compounds used in modern oral care products to interact with them. The special focus is on inorganic compounds and their interaction with oral biofilms. While organic compounds have several limitations (e.g., cell toxicity), inorganic compounds based on calcium and/or phosphate (e.g., sodium bicarbonate, hydroxyapatite, calcium carbonate) offer several advantages when used in oral care products. Calcium release can inhibit demineralization, and the release of hydroxide and phosphate ions might help in the buffering of acids. Therefore, the focus of this review is to summarize the scientific background of further active ingredients that can be used for oral care formulations.

## 1. Introduction

Biofilms and microorganisms are ubiquitous, and they mostly live as commensals with human beings [1]. However, mutations can occur in every microorganism and also changes in the composition of a biofilm, leading to a shift of a former commensal to a pathogenic-dominated composition [1,2,3,4]. As evolutionary pressure also leads to continuous changes in the characteristics of microorganisms, the co-existence between humans and microorganisms is a steadily developing progress [4]. While previous studies even reported that microbial cells exceed human cells, recent studies show an equilibrium of human and microbial cells [5,6]. Nevertheless, these results represent the importance of microbial cells for humans [5,6].

The oral cavity of man is generally believed to be sterile at birth, but shortly after birth, it becomes colonized by oral microorganisms [7]. From then onward, significant alterations in these microorganisms occurs before (predentate) and after (dentate) tooth eruption. The cariogenic microorganisms, particularly *Streptococcus mutans*, have been shown to infect the oral cavity of an infant as early as 3 months of birth [7]. Among the oral microorganisms, the major organisms associated with caries in man are (on the genus level), (a) *Streptococcus* (particularly *S. mutans*), (b) *Lactobacillus*, (c) *Actinomyces*, and more recently emerging (d) *Bifidobacteria*, as well as (e) *Veillonella* and (f) *Prevotella* [8,9]. These organisms possess special characteristics that enable them to adhere well to the tooth surface, produce higher amounts of acid from fermentable sugars than other oral bacterial species, and survive better than other bacteria in an acid environment. Cariogenic biofilms play important roles in the overall health of patients. The development and proliferation of those biofilms can only occur when the teeth are not properly and frequently cleansed using a toothbrush and toothpaste. While oral biofilms develop in every human being, they only become cariogenic when fermentable sugars are consumed. As an end product of the sugar metabolism, acids are produced that dissolve the enamel and dentin [10]. If this process is not controlled, it leads to the manifestation of dental caries. However, biofilm confers several beneficial properties to the patients. Dental plaque biofilm can play a role to promote remineralization and inhibit demineralization by serving as a reservoir for the targeted delivery of active agents to the plaque–tooth interface, to be released during an acidic challenge [11]. Furthermore, biofilm may help to prevent colonization by exogenous, and often pathogenic, microorganisms [12].

The oral cavity is a habitat where certain microorganisms co-exist with human cells as commensals or pathogens. While microorganisms such as bacteria, viruses, fungi, and protozoa are present in the oral cavity, human cells have developed several mechanisms to fight pathogens in the oral cavity [13,14,15,16]. While single-cell microorganisms mostly do not pose any harm, biofilm formation is more crucial for human health, as biofilms can be resistant to antibiotics [17]. Biofilms comprise several microbial species, interacting with each other [14,18,19,20]. This interaction can either positively or negatively influence the biofilm growth. In short, biofilms are a mixture of several microbial species synthetizing EPS (extracellular polymeric substances such as proteins, glycolipids, glycoproteins, and extracellular DNA) that are able to protect the microbial community [1,21]. Biofilms preferably grow where shedding and mechanical forces would not inhibit their formation. In the oral cavity, besides mechanical forces such as toothbrushing and abrasive diet, one of the natural mechanisms that inhibit biofilm growth is the salivary flow and its composition [22,23,24]. Saliva contains organic and inorganic components. Organic compounds such as proteins or enzymes (e.g., lactoferrin and lysozyme) with antimicrobial properties can combat biofilm formation or inhibit the growth of an initial biofilm [25,26,27]. The main inorganic compounds in saliva that interfere with the virulence of biofilms, particularly cariogenic biofilms, are phosphate and bicarbonate ions [28,29,30,31,32,33]. Bicarbonate and phosphate ions in saliva buffer the acids produced from carbohydrates (e.g., sucrose) metabolized by the bacteria in biofilms [29,34,35]. Acid buffering prevents demineralization of the tooth tissues by the acids, thus preventing dental caries development [31]. Furthermore, dietary intake of probiotics has been shown to control oral biofilm formation and growth as well as mitigate against some of the virulence factors of bacteria in the biofilm [8,36,37]. Basically, oral biofilms that lead to dental caries can be controlled to a certain extend with the help of the above mechanisms [28]. However, due to the modern diet and many plaque stagnation niches in the oral habitat, additional measures need to be applied to prevent dental caries and other plaque-associated diseases [38]. It is important to note that active agents used in oral care products need to weaken the virulence or at best disrupt oral biofilms, because plaque-free teeth would not develop dental caries [39]. In addition to using toothbrushes, irrespective of hand brushes, powered oscillating toothbrushes, or powered sonic toothbrushes [24], a straightforward way could be the incorporation of active agents in toothpastes and mouth rinses that will penetrate and retain in the oral niches to prevent biofilm growth, or at least reduce acidic biofilms and its potential to demineralize dental hard tissues [28,30,31,40].

The aim of this review is to give an overview of active ingredients, besides the well-known remineralizing agents such as fluorides or hydroxyapatite that can be incorporated into oral care formulations to modify the cariogenic biofilms.

## 2. Materials and Methods

The databases “BASE”, “PubMed”, and “SciFinder” were used for the literature search on active compounds used in oral care. Search terms were the following: “Oral care AND/OR active compounds AND/OR biofilm modification”. After screening the 1527 results (by 12 July 2021) from the three databases’ results for eligibility, none of the listed references could be directly classified to oral care products. Consequently, this review is articulated as a narrative review.

## 3. Results

The formation of biofilms, including oral biofilms, is a continuous process on any surfaces that are in contact with liquid [20]. Dental caries can be prevented in two different ways: (a) inhibition and reduction of cariogenic biofilm development and growth (antimicrobials) [41,42,43,44,45] and/or (b) using agents that can either buffer the microbial acids or reduce the demineralization potential of the microbial acids [31,45,46]. In the oral cavity, biofilm formation starts with the non-specific adhesion of bacteria to the pellicle. The pellicle is a thin layer that contains mostly salivary proteins [47,48]. Mucins and statherins are the main components of the pellicle to which bacteria can adhere [49,50]. Bacterial colonization can be subdivided into early colonization, so-called bridge-colonization, and late colonization [14,51]. Early colonizers (i.e., *Streptococcus* spp. and *Actinomyces* spp.) adhere to the pellicle using their surface antigen (a protein called adhesin) to form the initial layer of the biofilm, without the involvement of carbohydrates (so-called initial attachment phase) [13,52,53]. With the intake of a fermentable carbohydrate, the bacteria use its cell-bound and further extracelullar enzymes, mostly glucosyltransferases, to polymerize the glucose into adhesive extracellular carbohydrate polymers called glucan or extracellular polysaccharides (EPS). The bacteria use this adhesive EPS to bind to each other and to the tooth surfaces (so-called colonization phase) [53]. Thus, the biofilm is defined as a population of microorganisms growing on a surface, firmly attached to and enmeshed in an extracellular polysaccharide matrix [54]. As the oral cavity is a habitat for more than 700 different bacterial species, the bacterial colonization is followed by a biofilm growth phase [13,55]. The biofilm itself is a distinct ecosystem where different species and even taxa can positively or negatively influence each other, respectively [14,18,56,57]. Microorganisms can communicate by quorum sensing [57,58]. Quorum sensing is a mechanism where microorganisms produce certain molecules that can induce cell functions (e.g., a motility) in recipient cells when a certain threshold (depending on the bacterial strain and quorum-sensing molecule) has been reached [57,59]. Additionally, the EPS, produced by several microorganisms in biofilms, form a protective surrounding around the microbial colonies [1,17,60]. As the oral cavity is a small microcosm on its own and as the biofilm growth follows the rules of ecological pressure, it follows that when a certain kind of nutrition is available, the microorganisms that are best adapted to this food have an advantage over those that are not. Consequently, biofilms can change their composition and characteristics whenever the respective diet changes [61]. Thus, the biofilm growth phase is referred to as the remodeling phase, which is characterized by early dominance by the genus *Streptococcus* followed by a shift toward more anaerobic and filamentous flora [53]. Hence, over time, the cariogenic biofilms will change, as cells are dying or changing their properties because of ecological pressure [62].

It is well known that cariogenic biofilms develop and proliferate when the diet is rich in fermentable carbohydrates. The cariogenic microorganisms such as *Streptococcus* species, especially *S. mutans*, are very efficient in utilizing sucrose. These microorganisms have two metabolic systems (phosphotransferase (PTS) and proton motive force (pmf) systems) that are self-regulated to optimize glucose uptake with varying concentrations (low or high) of sugar in oral environment [63]. The PTS is a high-affinity sugar uptake mechanism driven by phosphoenol pyruvate (PEP) and operates at neutral pH. It enables the bacteria to take up glucose under low extracellular glucose concentrations. The PMF is a low-affinity sugar uptake system activated by a proton motive force (pmf) that operates at low pH, and it enables glucose uptake under high extracellular glucose concentrations [63]. Biofilm has reduced oxygen capacity, so as a survival mechanism, microorganisms produce their energy via anaerobic glycolysis. Glycolysis leads to the metabolism of glucose to adenosine triphosphate (ATP), which is the energy source for the bacteria cell growth and metabolism [63]. Lactic acids and some other organic acids are produced as the by-products of this glucose metabolism, and they diffuse from the biofilm to the tooth surfaces [64]. The acids cause the demineralization of enamel at a pH ≤ 5.5, which is the critical pH for the dissolution of enamel [65]. When enamel (and dentin) are demineralized, calcium and phosphate ions are released from the tooth tissue [10].

Interestingly, biofilms can imbibe calcium, acting as a calcium source for the reduction of subsequent acid attack [31,66,67]. It is pertinent to mention that the mechanical removal of the oral biofilms through oral hygiene procedures (e.g., toothbrushing) does not remove all dental biofilm from the dental hard tissues [24,68]. Biofilms can remain subgingival, supragingival, approximal, occlusal pits and fissures, and on the mucosa close to the tooth [69]. These residual biofilms also imbibe the active ingredients such as calcium and phosphate from oral care formulations in addition to the products of tooth dissolution. Thus, oral biofilm can play a role to promote remineralization and inhibit demineralization by serving as a reservoir for the targeted delivery of active agents to the plaque–tooth interface, which are released during an acidic challenge [11]. Suffice to say, the active ingredients in oral biofilm interact with the biofilm, changing it from pathogenic to healthy (protective) biofilm.

## 4. Preventive Measures

### 4.1. Biomimetic Substances for pH-Buffering in Cariogenic Biofilms

#### 4.1.1. Overview

In addition to mechanical plaque removal with the help of a toothbrush and toothpaste that usually mostly contains antibacterial compounds (e.g., zinc ions) [28,70], another concept can be applied to combat cariogenic biofilms [46,70,71,72,73,74]. Such a strategy uses different basic substances to buffer an acidic plaque pH. An important prerequisite for the use of these substances in oral care formulations such as toothpastes and mouth rinses is their non-toxicity [75]. For example, strong bases such as sodium hydroxide (NaOH) (one molar solution has a pH = 14) should not be used because of their non-physiological pH value and its associated cell toxicity. Oral care formulations should be formulated with a pH range of 6.8–7.4, as this is the physiological pH of saliva [76]. Consequently, to date, there is only a limited number of potential buffering agents that can be used for oral care formulations (Table 1).

In oral care formulations, mainly amino acids (e.g., arginine, C_6_H_14_N_4_O_2_) [77,78], carbonates (e.g., calcium carbonate, CaCO_3_) [79,80], and calcium phosphates [29] (e.g., hydroxyapatite, Ca_5_(PO_4_)_3_(OH) [31]), casein phosphopeptide-amorphous calcium phosphate (Ca_x_(PO_4_)_y_ *n* · H_2_O) [81], and α-tricalcium phosphate (α-Ca_3_(PO_4_)_2_) [82] are used as active ingredients to act as a buffer in oral care formulations against cariogenic biofilms.

#### 4.1.2. Organic Substances Used in Oral Care Formulations

Different organic components are known to act as buffer for acids. However, organic systems based on carboxylic acids (R-COOH) or amines (R-NH_2_) are not suitable for the use as buffering agents in oral care formulations. Their pK_a_ values are either too low (carboxylic acids: 3–5) or too high (amines: 9–11).

Organic compounds that can be used in oral care formulations are amino acids. Amino acids have (at least) one carboxylic acid group and one amino group per molecule. Basic amino acids that may act as buffering agents in cariogenic biofilms are arginine (pK_a_ = 12.48) and lysine (pK_a_ = 10.54). The pH range of buffering is well known to be between pH = 8 and 10, as reported by Fitch et al. [83]. Thus, organic acids that are produced by cariogenic biofilms can principally be buffered by both arginine and lysine. Studies have also shown the buffering effect induced by the arginine metabolism (production of ammonia via the arginine deiminase pathway by certain bacteria) [77,78]. Even though arginine is already used in toothpaste formulations, there are limitations that need to be taken into account. Arginine and other basic amino acids are mostly protonated at pH = 7. This is important because most toothpaste formulations have a pH value around 7; thus, the buffering capacities of arginine are significantly reduced in a toothpaste. From a chemical viewpoint, the pH of such toothpaste formulations should be highly basic, i.e., pH > 10, where arginine could act as buffer for acids (H^+^ acceptor) [83]. Additionally, arginine is highly soluble in water [84]. This means that the amino acid might be less substantive and can be very quickly washed out from the oral cavity [73]. When used regularly, arginine may cause some unwanted side effects such as diarrhea [85].

Arginine can be seen as prebiotic ingredient in oral care products, as some bacteria are able to metabolize this amino acid, for instance *Porphyromonas gingivalis*. *P. gingivalis* is known as a periodontogenic pathogen because it uses arginine via the protein arginine deiminase pathway (PAD) for biofilm formation and the citrullination of arginine. It uses the peptidyl arginine deiminase for the conversion of arginine to citrulline. Citrullination is associated with several multifactorial diseases such as periodontitis or rheumatoid arthritis. Consequently, the addition of arginine to a biofilm with *P. gingivalis* could lead to an increase in inflammation [56,86].

In addition to pure amino acids, proteins and peptides with free NH_2_ groups may also have buffering effects. One of those molecules is urea. However, studies have shown a positive correlation between salivary urea concentrations and dental calculus as well as periodontitis [87]. Although arginine can be used to lower the pH of cariogenic biofilms, its use in oral care products should be critically examined due to its potential to increase inflammation.

#### 4.1.3. Inorganic Particles Used in Oral Care Formulations

Solids with buffering effects are, for example, calcium carbonate (buffering effect by carbonate ions) and calcium phosphates (buffering effect by phosphate ions) [29,31,80,82,88]. Shaw et al. have shown that a higher dental plaque calcium content is associated with less dental caries [89]. In the case of calcium carbonate (CaCO_3_), there are several chemical reactions that can occur under acidic conditions:

1. Reaction of the proton with anions on the solid surface:

2 CaCO_3_ (s) + 2 H^+^ (aq) → Ca(HCO_3_)_2_ (s) + Ca^2+^ (aq)

2. Dissolution of calcium hydrogen carbonate:

Ca(HCO_3_)_2_ (s) → Ca^2+^ (aq) + 2 HCO_3_^−^ (aq)

3. Protonation of hydrogen carbonate to carbonic acid:

HCO_3_^−^ (aq) + H^+^ → H_2_CO_3_ (aq)

4. Decomposition of carbonic acid into carbon dioxide and water:

H_2_CO_3_ (aq) → CO_2_ (aq) + H_2_O (l)

5. Outgassing of carbon dioxide if its solubility is exceeded:

CO_2_ (aq) → CO_2_ (g)

All these reactions are equilibrium reactions and generally reversible. They depend mainly on the concentration of the reactants, temperature, and pressure.

In addition to calcium carbonate, hydroxyapatite (Ca_5_(PO_4_)_3_(OH)) can also act as a buffering agent in cariogenic biofilms as reported by Cieplik et al. [31]. Unlike calcium carbonate, the mechanism of the buffering effect of hydroxyapatite is more complex because OH^−^ and PO_4_^3−^ both have buffering effects. A simplified chemical equation for the buffering of acids would be [31]:

Ca_5_(PO_4_)_3_(OH) + 7 H^+^ → 5 Ca^2+^ + 3 H_2_PO_4_^−^ + H_2_O

In the case of hydroxyapatite, not only calcium ions will be released but also phosphate ions, both of which contribute to tooth remineralization (unlike in the case of calcium carbonate) [30,31,90]. An important requirement for a buffering effect in cariogenic biofilms is the adhesion of those particles (e.g., hydroxyapatite) to the tooth surface and the incorporation into biofilms as demonstrated in different research studies [41,43,91,92,93,94]. Particulate hydroxyapatite can increase the pH value of cariogenic in vitro biofilms from about 4.3 to 4.8 [31]. Stronger buffering effects can be achieved with carbonated hydroxyapatite or calcium carbonate [88]. Thus, a carbonated hydroxyapatite or a combination of hydroxyapatite/calcium carbonate may not only buffer organic acids but also release calcium and phosphate ions for tooth remineralization [30,90,95,96].

Note that both calcium carbonate and calcium phosphates cannot be formulated with soluble fluoride compounds (e.g., NaF or C_27_H_60_F_2_N_2_O_3_ (amine fluoride)) in toothpastes or mouthwashes because of the precipitation of calcium fluoride, CaF_2_; i.e., an “inactivation” of fluoride would occur under the release of carbonate or hydroxide ions [71,97,98]:

2 F^−^ (aq) + CaCO_3_ (s) → CaF_2_ (s) ↓ + CO_3_^2−^ (aq)

and

F^−^ (aq) + Ca_5_(PO_4_)_3_OH (s) → Ca_5_(PO_4_)_3_F (s) + OH^−^ (aq)

However, this is not a limitation for the use of such mineral buffering agents, since recent studies show that fluoride-free toothpastes based on biomimetic hydroxyapatite are not inferior to fluoride toothpastes with regard to remineralization and caries prevention in situ and in vivo [99,100,101]. Additionally, calcium phosphates can be used in caries prevention in small children because they are safe if accidently swallowed and do not induce dental fluorosis or other side effects [75,101]. Unlike soluble active ingredients for oral care applications (e.g., fluorides or cetylpyridinium chloride), mineral particles such as hydroxyapatite can occlude dentin tubules, resulting in a reduction of symptoms of dentin hypersensitivity [30,102,103,104,105,106,107].

### 4.2. Biomimetic Substances Modifying Bacterial Attachment to Tooth Surfaces

In addition to the release of calcium and phosphate ions and moderate pH-buffering properties in cariogenic biofilms [31], biomimetic hydroxyapatite can significantly reduce bacterial attachment to tooth surfaces without killing bacteria, as demonstrated in in situ studies [41,44]. In contrast, another calcium phosphate, i.e., casein phosphopeptide-amorphous calcium phosphate (CPP-ACP) does not lead to a reduction of initial biofilm formation in situ [81]. Interestingly, fluorides without an antibacterial counterion (NaF, Na_2_PFO_3_) do not minimize bacterial colonization [108]. This also supports the applicability of hydroxyapatite in oral biofilm control [41,44].

### 4.3. Probiotics

Probiotics are bacteria that are able to live and perform cell proliferation for growth [37,109]. Probiotic formulations should, at least, contain 10^6^ CFU mL^−1^ of live probiotic bacteria when being used [110]. As oral care products such as toothpastes and mouth washes are classified as cosmetic products in the EU, the use of living microorganisms can be critical [111]. However, other product classes are available such as medical devices. Probiotics, such as *Lacobacilli* spp., have been shown to positively influence caries biofilms [112,113]. As bacteria are producing so-called bacteriocins, *Lactobacilli* strains that are not cariogenic, but fighting acidogenic bacteria, can be used for oral care formulations. It is important to note that the use, as with all other oral care products, needs to be on a regular basis so that the oral homeostasis can be achieved [14].

### 4.4. Summary

Oral biofilms are ubiquitous and can develop in every individual. While change of the diet to less fermentable carbohydrates and the mechanical removal of dental plaque will reduce the potential of those biofilms to become cariogenic, active ingredients in oral care formulations can also help to reduce the cariogenicity of the biofilm in various ways, including but not limited to buffering the acidity. Scientific studies show biomimetic approaches using calcium- and carbonate-containing actives as promising ingredients that can be used to change the properties of cariogenic biofilms.

## 5. Conclusions

Cariogenic biofilms produce organic acids that can demineralize the tooth mineral. Consequently, the mechanical removal of these biofilms is essential. However, a complete removal of dental plaque is not possible in the daily oral care routine; therefore, active ingredients are needed that can incorporate into biofilms, to help in reducing the adverse effects of the acids produced by the bacteria. Basic amino acids have only a limited buffering effect due to its marginal adhesion to the tooth surface and rapid clearance from the oral cavity. In contrast to this, oral care formulations with buffering agents such as calcium carbonate and/or (carbonated) hydroxyapatite can interact with the tooth surface and have the potential to reduce the demineralization by cariogenic biofilms due to their buffering properties and potential calcium and phosphate ion release.

## Figures and Tables

**Table 1:** Overview of buffering systems for use under physiological conditions. The optimum buffering conditions occur when the pH is at the pKa value.

Buffer Systems	pK_a_ Values
B(OH)_3_/B(OH)_4_^−^ (borate buffer)	9.25
NH_3_/NH_4_^+^ (ammonium buffer)	9.24
HPO_4_^2−^/H_2_PO_4_^−^ (phosphate buffer)	7.21 (other pK_a_ values 2.16; 12.32)
Citric acid/citrate	3.13; 4.76; 6.4
Carbonic acid/hydrogen carbonate	6.1
CH_3_COO^−^/CH_3_COOH	4.76

## Data Availability

Not Applicable.
